# Impaired nuclear import and viral incorporation of Vpr derived from a HIV long-term non-progressor

**DOI:** 10.1186/1742-4690-5-67

**Published:** 2008-07-18

**Authors:** Leon Caly, Nitin K Saksena, Sabine C Piller, David A Jans

**Affiliations:** 1 Department of Biochemistry and Molecular Biology, Monash University, Clayton, Victoria 3800, Australia; 2 Retroviral Genetics Division, Centre for Virus Research, Westmead Millennium Institute, Westmead Hospital, The University of Sydney, Darcy Road, Westmead, N.S.W 2145, Australia; 3 HIV Protein Function and Interactions Group, Centre for Virus Research, Westmead Millennium Institute, Westmead Hospital, The University of Sydney, Darcy Road, Westmead, N.S.W 2145, Australia; 4 University of Western Sydney, Penrith South DC, N.S.W 1797, Australia

## Abstract

We previously reported an epidemiologically linked HIV-1 infected patient cohort in which a long-term non-progressor (LTNP) infected two recipients who then exhibited normal disease progression. Expression of patient-derived *vpr *sequences from each of the three cohort members in mammalian cells tagged with GFP revealed a significant reduction in Vpr nuclear import and virion incorporation uniquely from the LTNP, whereas Vpr from the two progressing recipients displayed normal localisation and virion incorporation, implying a link between efficient Vpr nuclear import and HIV disease progression. Importantly, an F72L point mutation in the LTNP was identified for the first time as being uniquely responsible for decreased Vpr nuclear import.

## Findings

The highly conserved HIV accessory protein Viral Protein R (Vpr) is vital for HIV infection and replication *in vivo*, particularly within non-dividing cells, including terminally differentiated T-cells and macrophages where nuclear envelope integrity is permanently maintained [1-5]. On its own and within the context of HIV infection [6,7], Vpr has been shown to localize to the nucleus and induce G_2 _cell-cycle arrest through hyperphosphorylation of p34-cdc2, which provides the most optimal environment for viral replication [8-13], followed closely by apoptosis [14-21]. During HIV infection, Vpr associates with the viral cDNA containing Pre-integration Complex (PIC) increasing its affinity for components of the cellular nuclear import machinery [22-25] through the activity of 2 distinct nuclear localization signals (NLSs) within its N- [26] and C-termini [27], thus driving productive HIV infection.

We previously reported [28] on a cohort spanning 1992 to 2000, comprising an HIV^+ ^long-term non-progressor (LTNP) (donor A) and two recipients (B and C) who upon transmission (autumn and summer 1989 respectively) from donor A progressed to AIDS. *Vpr *sequences derived from HIV pro-viral DNA isolated from PBMCs as well as circulating virus, revealed that sequences from the progressing recipients differed markedly to those of the LTNP founder virus host, providing the first evidence for Vpr positive selection during disease progression in an epidemiologically-linked cohort (see also [29]).

**Figure 1:**
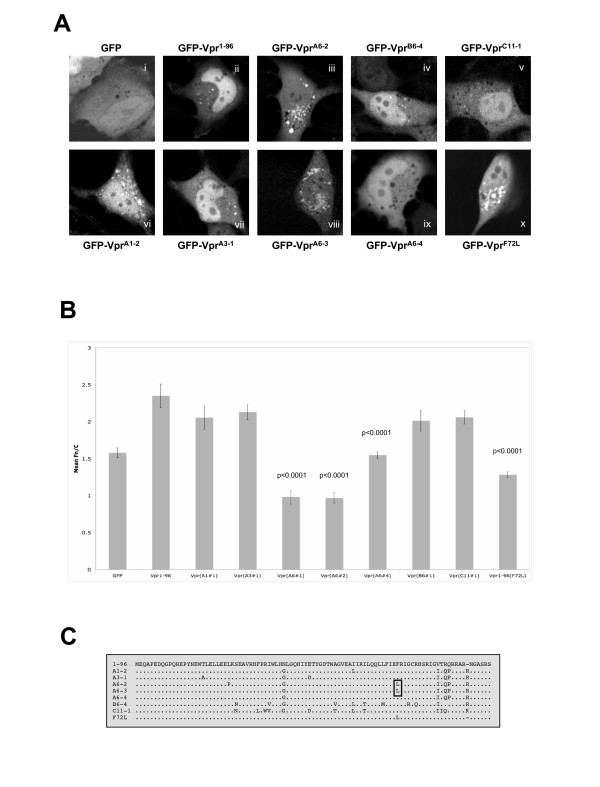
**Late-stage LTNP-derived GFP-Vpr samples show decreased levels of nuclear accumulation compared to wildtype Vpr and progressing donors B and C**. **(A) **HeLa cells were transfected with indicated GFP-Vpr constructs using Lipofectamine 2000™ with confocal images obtained 14 hours later using an Olympus FV1000 CLSM. **(B) **Analysis of CLSM images (as per **A**) with ImageJ was performed to determine the Fn/c. As a whole, late-stage samples from patient A (A6-2, A6-3, A6-4) and the Vpr (F72L) point mutant displayed significantly (p < 0.0001) reduced Fn/c ratios compared to wildtype Vpr. **(C) **Sequence analysis of patient-derived *vpr *sequences identifies F72L substitution mutation (as indicated) uniquely within all GFP-Vpr constructs with reduced nuclear accumulation.

In this study, cohort-derived GFP-Vpr mammalian cell expression vectors were used to investigate Vpr subcellular localisation and nuclear import. In total, 4 HIV-1 *vpr *clones were isolated from donor A (A1, A3, A5 and A6 corresponding to years 1996, 1998, 1999 and 2000), 4 from recipient B (B3, B4, B5 and B6 corresponding to years 1997, 1998, 1998 and 1999) and 7 from recipient C (C2, C3, C5, C6, C8, C10 and C11 from 1992, 1992, 1993, 1994, 1994, 1998 and 1999 respectively) and used to generate the GFP-Vpr expression constructs. Lipofectamine™ 2000 (Invitrogen) was used to transfect constructs into HeLa and COS-7 (*not shown*) cells, followed by imaging 14 hours post transfection using an Olympus FV-1000 confocal laser scanning microscope (CLSM), with similar results. Initial analysis of late-stage *vpr *gene products from all 3 donors revealed a reduction in nuclear fluorescence accompanied by perinuclear accumulation for LTNP sample A6-2 (patient **A**, time-point **6**, clone **#2**) (Fig. 1A(iii)) compared to pUC18-NL4.3 derived [30] wild-type GFP-Vpr^1–96 ^(Fig. 1A(ii)): a phenotype not observed in samples from patients B (B6-4) nor C (C11-1) (Fig 1A(iv, v)). Quantification of nuclear import was determined using ImageJ analysis software [31] where the ratio of nuclear (Fn) to cytoplasmic (Fc) fluorescence, subtracting background (B) was calculated, Fn/c = (Fn-B)/(Fc-B). A highly significant (p < 0.0001) reduction in Fn/c was observed between GFP-Vpr^1–96 ^(Fn/c ~2.4) and GFP-Vpr^A6-2 ^(Fn/c ~0.9) indicating a reduction in nuclear accumulation. No significant difference in Fn/c was observed between GFP-Vpr^1–96 ^and GFP-Vpr^B6-4 ^or GFP-Vpr^C11-1 ^(Fig. 1B).

To establish if decreased Vpr nuclear accumulation was a specifically conserved viral feature of the LTNP or an acquired trait, GFP-Vpr constructs from early-cohort timepoints were analysed. Samples from 1996 (GFP-Vpr^A1-2^) and 1998 (GFP-Vpr^A3-1^) (Fig. 1A(vi, vii)) both displayed levels of nuclear accumulation comparable to wildtype GFP-Vpr^1–96 ^(Fig. 1B), implying that loss of nuclear import occurred post 1998.

With the loss of efficient nuclear accumulation identified as a late-stage LTNP trait, further analysis of late-stage clones A6-3 and A6-5 (*clone A6-5 data not shown as sequence is homologous to A6-3*) was performed, revealing Fn/c values significantly lower than wildtype GFP-Vpr^1–96 ^(Fig. 1B). Of interest, a single late-stage clone (GFP-Vpr^A6-4^) displayed a small degree of nuclear accumulation (Fig. 1A(ix)), although at levels significantly reduced compared to wildtype (Fig. 1B). Sequence analysis revealed that all clones with reduced nuclear accumulation (Vpr^A6-2 ^and Vpr^A6-3/5^) contained a phenylalanine to leucine mutation at amino acid position 72 (F72L) (Fig. 1C). This mutation is absent in the nuclear localizing Vpr^A6-4^, which is otherwise identical in sequence to late-stage samples Vpr^A6-3/5^. Analysis of our database containing over 1200 published and unpublished Vpr sequences revealed that the F72L mutation has only been reported once previously [32].

To determine the specific role of F72L in abrogation of Vpr nuclear import, an F72L substitution was engineered into wildtype Vpr and an expression construct (GFP-Vpr^F72L^) produced. Subsequent transfection (Fig. 1A(x)) revealed a reduction in GFP-Vpr^F72L ^nuclear accumulation when compared with wildtype GFP-Vpr^1–96 ^(Fig. 1A(ii)), in a similar fashion to clinically derived GFP-Vpr^A6-2 ^(Fig. 1B), thus confirming F72L as a probable mechanism for the observed reduction in nuclear accumulation within late-stage LTNP samples. This reduction in nuclear import was accompanied by an accumulation of GFP-Vpr protein within the perinuclear region of the cell, concordant with an area encompassing the proposed location of the Golgi. Colocalisation studies with the Golgi-specific marker protein γ-adaptin revealed that Vpr proteins harbouring the F72L point mutation, but not those with wildtype phenylalanine, were localized to the Golgi apparatus, implying that reduced nuclear accumulation could in part be attributed to this mislocalisation of GFP-Vpr (see Additional file 1). The lack of colocalisation between F72L-containing Vpr proteins and the endoplasmic reticulum (ER) marker Calnexin (Sigma, C-4731), confirmed that Vpr mislocalisation to the Golgi is not a result of general mistargeting (see Additional file 2).

**Figure 2:**
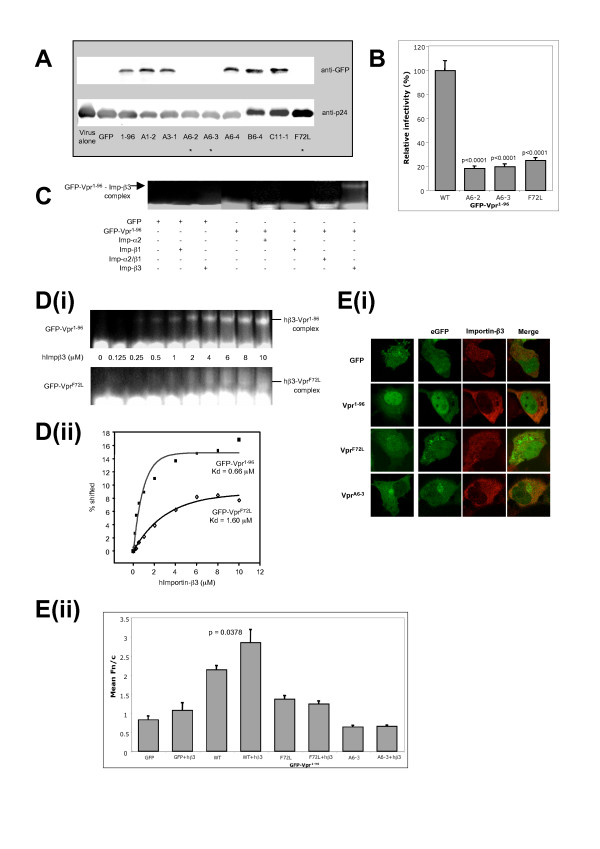
**F72L-containing GFP-Vpr proteins fail to incorporate into forming virions, which show decreased infectivity of non-dividing MAGI (Multinuclear Activation of a Galactosidase Indicator) cells correlating with reduced importin-β3 binding**. **(A**) Virus was derived from 293T cells cotransfected with pEPI-GFP-Vpr and proviral plasmid pUC18-NL4.3 and subjected to Western blot analysis, revealing the absence of GFP-Vpr protein in F72L containing samples. Control staining for p24 capsid protein indicates the presence of virus in all samples (*denotes lack of virion incorporation). **(B) **Virus derived from 293T cells cotransfected with the ΔVpr pro-viral plasmid pUC18-NL4.3(FS) and pEPI-GFP-Vpr (1–96, A6-2, A6-3 or F72L) was purified, normalized using an RT assay and used to infect growth arrested (γ-irradiated, 2 cycles at 30 Gy) MAGI (CD4^+^, integrated HIV-1-LTR-β-gal) cells. 48 hours post infection cells were fixed (1% formaldehyde/0.2% glutaraldehyde/PBS), stained (4 mM potassium ferricyanide, 4 mM potassium ferrocyanide, 2 mM MgCl_2_, 0.4 mg/ml 5-bromo-4-chloro-3-indolyl-β-D-galactopyranoside [X-gal]) and scored for viral infectivity. Infected cells display X-gal stained (blue) nuclei due to expression of the early HIV protein Tat, which binds the HIV-LTR promoter of the integrated β-gal gene, resulting in expression of the β-gal protein. Non-infected cells remain colourless due to the lack of Tat expression and subsequent activation of the β-gal gene. Data presented depicts relative levels of infectivity (% +/- SEM) compared to wildtype Vpr^1–96^. Virus derived from F72L-cotransfected 293T cells displayed a significant (p < 0.0001) 5-fold reduction in viral infectivity of non-dividing cells compared to wildtype Vpr^1–96^. **(C) **Native PAGE gel-shift mobility assay; 2 μM GFP-Vpr^1–96 ^or GFP alone was incubated with 10 μM importin-α2, -β1, -α2/β1 or -β3 as indicated. **(D) **Native PAGE gel-shift mobility titration assay; (i) 2 μM GFP-Vpr^1–96 ^or GFP-Vpr^F72L ^was incubated with increasing concentrations of Importin-β3 protein as indicated. (ii) Fluorimetric analysis of gel-shift assays from D(i), was performed as per [39] with the binding curves generated used to calculate dissociation constants (*K*_d_). **(E)i **Typical CLSM images of fixed COS-7 cells expressing the indicated GFP-fusion proteins alone or in the context of c-myc-tagged-human Importin-β3. Cells were permeabilised and stained 14 hours post transfection with anti-c-myc antibody (Sigma) and visualized with Alexa-Fluo-568 (Molecular Probes). **(E)ii **Analysis of CLSM images (as per **E(i)**) with ImageJ was performed to determine the Fn/c. Exogenous Importin-β3 was found to significantly (p = 0.0378) increase the nuclear accumulation of wildtype GFP-Vpr^1–96^, but not that of GFP-Vpr^F72L ^or F72L containing GFP-Vpr^A6-3^.

Although the precise pathways involved are still debated, efficient nuclear export [33] and/or cytoplasmic retention [34] of Vpr during viral infection has been identified as a key requirement for virion incorporation. With this in mind, we assessed the ability of nuclear import-inhibited cytosolic GFP-Vpr^F72L ^to incorporate into forming virions. Briefly, 293T cells were cotransfected with a panel of GFP-Vpr expression vectors as well as the proviral plasmid pUC18-NL4.3, with the resultant virions analysed by Western blotting for GFP-Vpr incorporation. Interestingly we found that all F72L-containing GFP-Vpr proteins that failed to significantly localize within the nucleus, were absent from purified viral lysates (Fig. 2A), implying a possible link between the efficient nuclear transport of Vpr and virion incorporation. To examine the effect of the F72L mutation on HIV infectivity, we infected γ-irradiated, non-dividing CD4^+ ^MAGI (Multinuclear Activation of a Galactosidase Indicator) cells which contain an integrated HIV-1-LTR-β-galactosidase gene with virus derived from 293T cells cotransfected with ΔVpr pro-virus and wildtype or F72L-containing GFP-Vpr encoding plasmids. 48 hours post infection, MAGI cells were fixed and stained with 5-bromo-4-chloro-3-indolyl-beta-Dgalactopyranoside (X-gal) [35], followed by scoring for infected cells (blue nuclei) due to LTR-β-gal transactivation by viral Tat protein. Virus derived from all F72L-containing plasmids showed a significant (p < 0.0001), 5-fold reduction in viral infectivity when compared to wildtype Vpr^1–96 ^(Fig. 2B). This dramatic reduction in infectivity presumably stems from the lack of virion incorporation of F72L-containing Vpr (Fig. 2A) and resultant absence of Vpr-facilitated PIC nuclear import, a key factor in efficient HIV replication within non-dividing cells [1,24,25,36].

Nuclear accumulation of Vpr is governed by 2 distinct NLSs; a leucine-rich N-terminal α-helix (^20^LELLEEL^26^) which has been linked to virion incorporation [37,38], and a C-terminal arginine-rich bipartite (underlined) (^71^H**F**RIGCRHSRIGVTRQRRAR^90^) NLS [27] which incorporates phenylalanine 72 (bold). To determine if the identified F72L substitution affects the function of Vpr's C-terminal NLS, we used a previously described fluorescence based gel-shift mobility assay [39] to compare the interaction between wildtype GFP-Vpr^1–96 ^and GFP-Vpr^F72L ^with a panel of purified importin proteins. We established that wildtype GFP-Vpr^1–96 ^was able to bind with high affinity to human Importin-β3 (hβ3) but not other members of the importin superfamily, including Importin-β1, Importin-α2 or the Importin-α2/β1 heterodimer. (Fig. 2C). Titrations revealed that GFP-Vpr^F72L ^was unable to bind hβ3 as effectively as wildtype GFP-Vpr^1–96 ^(K_d _= 0.66 μM vs. 1.60 μM) (Fig. 2D(i, ii)), indicating a direct relationship between F72L and decreased Importin-β3 binding capacity. *In vivo *cotransfection of GFP-Vpr constructs with Importin-β3 was found to increase the Fn/c of wildtype GFP-Vpr^1–96^, but not that of GFP-Vpr^F72L ^or GFP-Vpr^A6-3^, consistent with the idea that Importin-β3 contributes to nuclear import of Vpr, and that the F72L mutation impacts on recognition by Importin-β3 directly (see Fig. 2E(i, ii)). We therefore propose that the F72L mutation itself leads to decreased Vpr nuclear import and consequently reduced virion incorporation due to reduced binding efficiency to Importin-β3.

It does not seem unreasonable to speculate that *in vivo *selective pressures have driven the evolution of functionally-attenuated *vpr *to ensure long-term viral survival and replication *in vivo *[2,5,40-42]. Our study shows that F72L-containing Vpr does not accumulate within host cell nuclei or incorporate into nascent virions; subsequent infections with such a Vpr-deficient virus are less efficient, leading to reduced virus production [41-43] and ultimately a lack of deterioration in T-cell numbers, as in the case of the LTNP. Many of our observations are concordant with analysis of LTNP infection status in 2000, which revealed normal CD4^+ ^T-cell levels (>550 cells per μl) suggesting the absence of significant HIV-mediated T-cell targeting and depletion. Since our data here suggests that c. 75% of late-stage proviral strains in the LTNP would produce virions lacking Vpr due to the F72L mutation, the levels of observed CD4^+ ^T-cells within the LTNP may be due to the inability of HIV lacking Vpr to import the viral genome into the non-dividing host cell nucleus and carry out subsequent steps of infection [1,24,25,44]. Presumably one advantage for the virus is that the preservation of patient health status leads to enhanced probability of transmission to new hosts, thus leading to increased virus spread. Low levels of virus production (<425 HIV copies/ml plasma) [29] are presumably maintained through the activity of a small subset of Vpr quasi-species that do not contain the F72L mutation (A6-4) or perhaps viruses that we were unable to characterize due to their low prevalence. Additional to all of the above considerations, it is important to remember that the effects of decreased Vpr nuclear import through the F72L mutation are likely to be influenced by many other factors, including host genetic make-up and immune response, which in themselves can influence disease progression [28,45-49].

In summary this study provides evidence for a naturally occurring Vpr mutation within an epidemiologically-linked cohort and its possible contribution to non-progressive HIV disease status through disruption of efficient nuclear transport and apparent lack of viral incorporation, leading to reduced infectivity of non-dividing cells (Fig. 2B). To demonstrate formally the link between Vpr nuclear localisation and disease progression, further analysis of Vpr^F72L ^in a HIV infectious system is required, eg. to dismiss the possibility that the effects in terms of lack of assembly of Vpr^F72L ^may in part be due to impaired p6gag binding, stemming from conformational or other effects. With this proviso in mind, however, the work here and elsewhere (see also [50]) implies that functional selection of *vpr *viral quasispecies in concert with host selection pressure over time, as evident in the LTNP, may be a factor in determining the rate of disease progression.

## Supplementary Material

Additional file 1 **LTNP derived Vpr proteins with reduced nuclear accumulation localize within the Golgi**. Typical CLSM images of fixed COS-7 cells expressing the indicated GFP-fusion proteins. Cells were permeabilised and stained 14 hours post transfection for γ-adaptin and visualized with Alexa-Fluor-568. GFP-Vpr proteins specifically containing F72L show colocalisation with the Golgi apparatus as indicated by arrows. Click here for file

Additional file 2 **LTNP derived Vpr proteins with reduced nuclear accumulation localize within the Golgi**. Typical CLSM images of fixed COS-7 cells expressing the indicated GFP-fusion proteins. Cells were permeabilised and stained 14 hours post transfection for Calnexin and visualized with Alexa-Fluor-568. GFP-Vpr was found to not localize within the ER. Click here for file
