# Supplementary material for: Impaired nuclear import and viral incorporation of Vpr derived from a HIV long-term non-progressor
Source: Retrovirology. 2008 Jul 18;5:67. doi: 10.1186/1742-4690-5-67 (PMC2515335; doi:10.1186/1742-4690-5-67)
Supplement: Additional file 2 — LTNP derived Vpr proteins with reduced nuclear accumulation localize within the Golgi. Typical CLSM images of fixed COS-7 cells expressing the indicated GFP-fusion proteins. Cells were permeabilised and stained 14 hours post transfection for Calnexin and visualized with Alexa-Fluor-568. GFP-Vpr was found to not localize within the ER. [file 1742-4690-5-67-S2.ppt]

## Slide 1
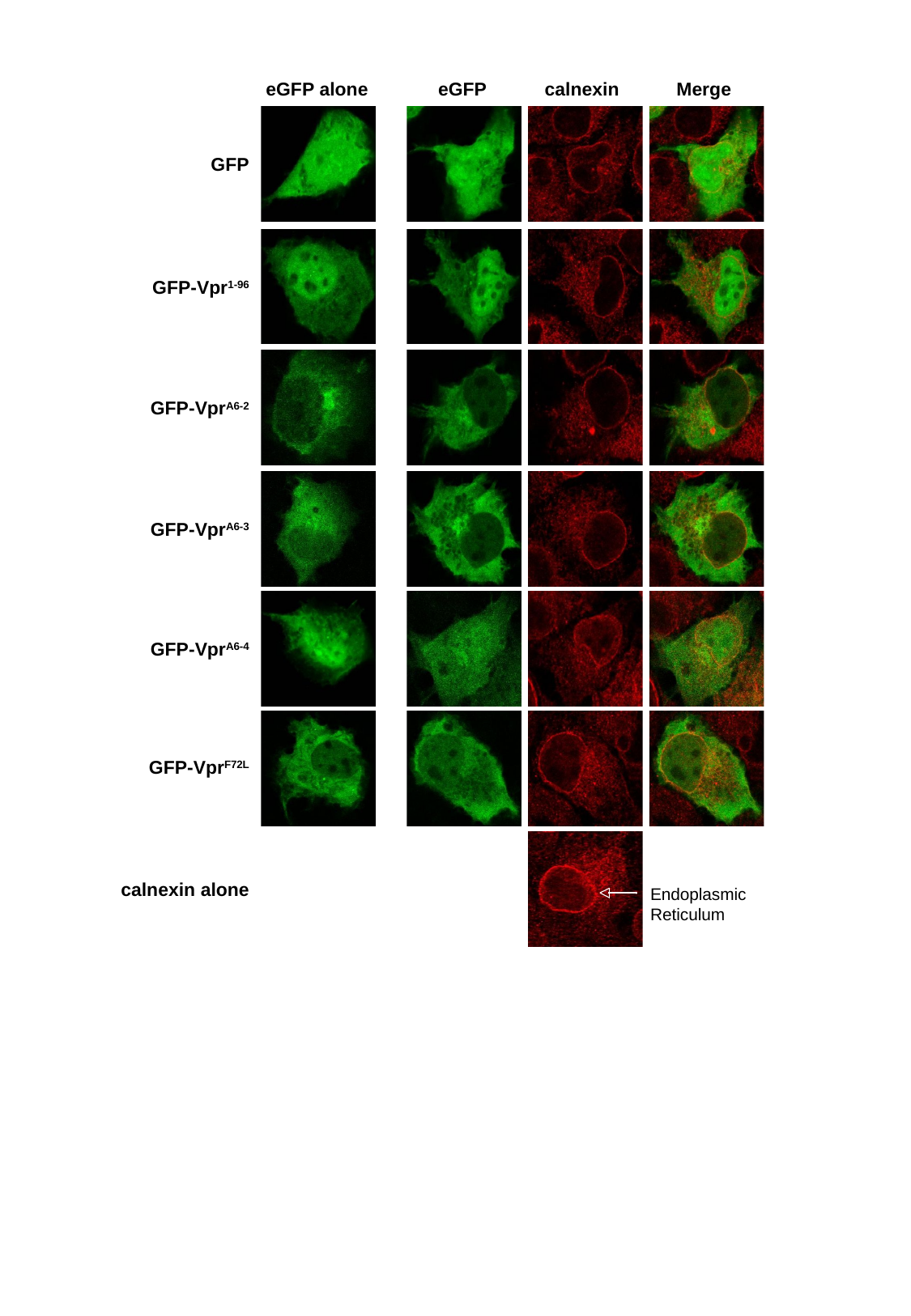

eGFP alone
eGFP
calnexin
Merge
GFP
GFP-Vpr1-96
GFP-VprA6-2
GFP-VprA6-3
GFP-VprA6-4
GFP-VprF72L
calnexin alone
Endoplasmic
Reticulum
